# Thalamocortical contribution to flexible learning in neural systems

**DOI:** 10.1162/netn_a_00235

**Published:** 2022-10-01

**Authors:** Mien Brabeeba Wang, Michael M. Halassa

**Affiliations:** Department of Brain and Cognitive Science, Massachusetts Institute of Technology, Cambridge, MA, USA; Computer Science and Artificial Intelligence Laboratory, Massachusetts Institute of Technology, Cambridge, MA, USA

**Keywords:** Meta-learning, Credit assignment, Continual learning, Thalamocortical interactions, Basal ganglia, Thalamus

## Abstract

Animal brains evolved to optimize behavior in dynamic environments, flexibly selecting actions that maximize future rewards in different contexts. A large body of experimental work indicates that such optimization changes the wiring of neural circuits, appropriately mapping environmental input onto behavioral outputs. A major unsolved scientific question is how optimal wiring adjustments, which must target the connections responsible for rewards, can be accomplished when the relation between sensory inputs, action taken, and environmental context with rewards is ambiguous. The credit assignment problem can be categorized into context-independent *structural credit assignment* and context-dependent *continual learning*. In this perspective, we survey prior approaches to these two problems and advance the notion that the brain’s specialized neural architectures provide efficient solutions. Within this framework, the thalamus with its cortical and basal ganglia interactions serves as a systems-level solution to credit assignment. Specifically, we propose that thalamocortical interaction is the locus of meta-learning where the thalamus provides cortical control functions that parametrize the cortical activity association space. By selecting among these control functions, the basal ganglia hierarchically guide thalamocortical plasticity across two timescales to enable meta-learning. The faster timescale establishes contextual associations to enable behavioral flexibility, while the slower one enables generalization to new contexts.

## Author Summary

Deep learning has shown great promise over the last decades, allowing artificial neural networks to solve difficult tasks. The key to success is the optimization process by which task errors are translated to connectivity patterns. A major unsolved question is how the brain optimally adjusts the wiring of neural circuits to minimize task error analogously. In our perspective, we advance the notion that the brain’s specialized architecture is part of the solution and spell out a path towards its theoretical, computational, and experimental testing. Specifically, we propose that the interaction between the cortex, thalamus, and basal ganglia induces plasticity in two timescales to enable flexible behaviors. The faster timescale establishes contextual associations to enable behavioral flexibility, while the slower one enables generalization to new contexts.

## INTRODUCTION

Learning to flexibly choose appropriate actions in uncertain environments is a hallmark of intelligence (Miller & Cohen, 2001; Niv, 2009; Thorndike, 2017). When animals explore unfamiliar environments, they tend to reinforce actions that lead to unexpected rewards. A common notion in contemporary neuroscience is that such behavioral reinforcement emerges from changes in synaptic connectivity, where synapses that contribute to the unexpected reward are strengthened (Abbott & Nelson, 2000; Bliss & Lomo, 1973; Dayan & Abbott, 2005; Hebb, 2002; Whittington & Bogacz, 2019). A prominent model for connecting synaptic to behavioral reinforcement is dopaminergic innervation of basal ganglia (BG), where dopamine (DA) carries the **reward prediction error** (RPE) signals to guide synaptic learning (Bamford, Wightman, & Sulzer, 2018; Bayer & Glimcher, 2005; Montague, Dayan, & Sejnowski, 1996; Schultz, Dayan, & Montague, 1997). This circuit motif is thought to implement a basic form of the reinforcement learning algorithm (Houk, Davis, & Beiser, 1994; Morris, Nevet, Arkadir, Vaadia, & Bergman, 2006; Roesch, Calu, & Schoenbaum, 2007; Suri & Schultz, 1999; R. Sutton & Barto, 2018; R. S. Sutton & Barto, 1990; Wickens & Kotter, 1994), which has had much success in explaining simple Pavlovian and instrumental conditioning (Ikemoto & Panksepp, 1999; Niv, 2009; R. Sutton & Barto, 2018; R. S. Sutton & Barto, 1990). However, it is unclear how this circuit can reinforce the appropriate connections in complex natural environments where animals need to dynamically map sensory inputs to different action in a context-dependent way. If one naively credits all synapses with the RPE signals, the learning will be highly inefficient since different cues, contexts, and actions contribute to the RPE signals differently. To properly credit the cues, context, and actions that lead to unexpected reward is a challenging problem, known as the **credit assignment** problem (Lillicrap, Santoro, Marris, Akerman, & Hinton, 2020; Minsky, 1961; Rumelhart, Hinton, & Williams, 1986; Whittington & Bogacz, 2019).

One can roughly categorize the credit assignment into context-independent *structural credit assignment* and context-dependent ***continual learning***. In structural credit assignment, animals may make decisions in a multi-cue environment and should be able to credit those cues that contribute to the rewarding outcome. Similarly, if actions are being chosen based on internal decision variables, then the underlying activity states must also be reinforced. In such cases, neurons that are selective to external cues or internal latent variables need to adjust their downstream connectivity based on its contribution of their downstream targets to the RPE. This is a challenging computation to implement because, for upstream neurons, the RPE will be dependent on downstream neurons that are several connections away. For example, a sensory neuron needs to know the action chosen in the motor cortex to selectively credit the sensory synapses that contribute to the action. In *continual learning*, animals not only need to appropriately credit the sensory cues and actions that lead to the reward but also need to credit the sensorimotor combination in the right context to retain the behaviors learned from different contexts and even to generalize to novel contexts. Therefore, animals can continually learn and generalize across different contexts while retaining behaviors in familiar contexts. For example, when one is in the United States, one learns to first look left before crossing the street, whereas in the United Kingdom, one learns to look right instead. However, after spending time in the United Kingdom, someone from the United States should not unlearn the behavior of looking left first when they return home because their brain ought to properly assign the credit to a different context. Furthermore, once one learns how to cross the street in the United States, it is much easier to learn how to cross the street in the United Kingdom because the brain flexibly generalize behaviors across contexts.

In this perspective, we will first go over common approaches from machine learning to tackle these two credit assignment problems. In doing so, we highlight the challenge in their efficient implementation within biological neural circuits. We also highlight some recent proposals that advance the notion of specialized neural hardware that approximate more general solutions for credit assignment (Fiete & Seung, 2006; Ketz, Morkonda, & O’Reilly, 2013; Kornfeld et al., 2020; Kusmierz, Isomura, & Toyoizumi, 2017; Lillicrap, Cownden, Tweed, & Akerman, 2016; Liu, Smith, Mihalas, Shea-Brown, & Sümbül, 2020; O’Reilly, 1996; O’Reilly, Russin, Zolfaghar, & Rohrlich, 2021; Richards & Lillicrap, 2019; Roelfsema & Holtmaat, 2018; Roelfsema & van Ooyen, 2005; Sacramento, Ponte Costa, Bengio, & Senn, 2018; Schiess, Urbanczik, & Senn, 2016; Zenke & Ganguli, 2018). Along these lines, we propose an efficient systems-level solution involving the thalamus and its interaction with the cortex and BG for these two credit assignment problems.

## COMMON MACHINE LEARNING APPROACHES TO CREDIT ASSIGNMENT

One solution to structural credit assignment in machine learning is **backpropagation** (Rumelhart et al., 1986). Backpropagation recursively computes the vector-valued error signal for synapses based on their contribution to the error signal. There is much empirical success of backpropagation in surpassing human performance in supervised learning such as image recognition (He, Zhang, Ren, & Sun, 2016; Krizhevsky, Sutskever, & Hinton, 2012) and reinforcement learning such as playing the game of Go and Atari (Mnih et al., 2015; Schrittwieser et al., 2020; Silver et al., 2016; Silver et al., 2017). Additionally, comparing artificial networks trained with backpropagation with neural responses from the ventral visual stream of nonhuman primates shows comparable internal representations (Cadieu et al., 2014; Yamins et al., 2014). Despite its empirical success in superhuman-level performance and matching the internal representation of actual brains, backpropagation may not be straightforward to implement in biological neural circuits, as we explain below.

In its most basic form, backpropagation requires symmetric connections between neurons (forward and backward connections). Mathematically, we can write down the backpropagation in Equation 1:

$$\delta W_{i}\propto \frac{\partial E}{\partial W_{i}}=e_{i}f{\left(a_{i-1}\right)}^{⊤},$$ (1)

where

$$e_{i}=W_{i+1}^{T}e_{i+1}\circ f^{\prime }\left(a_{i}\right),$$

*E* is the total error, *e*_*i*_ is the vector error at layer *i*, *W*_*i*_ is the synaptic weight connecting layer *i* − 1 to layer *i*, and *f* is the nonlinearity. Intuitively, this is saying that the change of synaptic weight *W*_*i*_ is computed by a Hebbian learning rule between backpropagation error *e*_*i*_ and activity from last layer *f*(*a*_*i*−1_), while the backpropagation error is computed by backpropagating the error in the next layer through symmetric feedback weights $W_{i+1}^{⊤}$. Importantly, in this algorithm, error signals do not alter the activity of neurons in the preceding layers and instead operate independently from the feedforward activity. However, such arrangement is not observed in the brain; symmetric connections across neurons are not a universal feature of circuit organization, and biological neurons may encode both feedforward inputs and errors through changes in spike output (changes in activity; Crick, 1989; Richards & Lillicrap, 2019). Therefore, it is hard to imagine how the basic form of backpropagation (symmetry and error/activity separation) is physically implemented in the brain.

Furthermore, while an animal can continually learn to behave across different contexts, artificial neural networks trained by backpropagation struggle to learn and remember different tasks in different contexts: a problem known as **catastrophic forgetting** (French, 1999; Kemker, McClure, Abitino, Hayes, & Kanan, 2018; Kumaran, Hassabis, & McClelland, 2016; McCloskey & Cohen, 1989; Parisi, Kemker, Part, Kanan, & Wermter, 2019). Specifically, this problem occurs when the tasks are trained sequentially because the weights optimized for former tasks will be modified to fit the later tasks. One of the common solutions is to interleave the tasks from different contexts to jointly optimize performance across contexts by using an episodic memory system and replay mechanism (Kumaran et al., 2016; McClelland, McNaughton, & O’Reilly, 1995). This approach has received empirical success in artificial neural networks, including learning to play many Atari games (Mnih et al., 2015; Schrittwieser et al., 2020). However, since one needs to store past training data in memory to replay during learning, this approach demands a high computational overhead and can be is inefficient as the number of the contexts increases. On the other hand, humans and animals acquire diverse sensorimotor skills in different contexts throughout their life span: a feat that cannot be solely explained by memory replay (M. M. Murray, Lewkowicz, Amedi, & Wallace, 2016; Parisi et al., 2019; Power & Schlaggar, 2017; Zenke, Gerstner, & Ganguli, 2017). Therefore, biological neural circuits are likely to employ other solutions to continual learning in addition to memory replay.

Therefore, to solve these two credit assignment problems in the brain, one needs to seek different solutions. One of the pitfalls of backpropagation is that it is a general algorithm that works on any architecture. However, actual brains are collections of specialized hardware put together in a specialized way. It can be conceived that through clever coordination between different cell types and different circuits, the brains can solve the credit assignment problem by leveraging its specialized architectures. Along this line of ideas, many investigators have proposed cellular (Fiete & Seung, 2006; Kornfeld et al., 2020; Kusmierz et al., 2017; Liu et al., 2020; Richards & Lillicrap, 2019; Sacramento et al., 2018; Schiess et al., 2016) and circuit-level mechanisms (Lillicrap et al., 2016; O’Reilly, 1996; Roelfsema & Holtmaat, 2018; Roelfsema & van Ooyen, 2005) to assign credit appropriately. In this perspective, we would like to advance the notion that the specialized hardware arrangement also happens at the system level and propose that the thalamus and its interaction with basal ganglia and the cortex serve as a system-level solution for these three types of credit assignment.

## A PROPOSAL: THALAMOCORTICAL–BASAL GANGLIA INTERACTIONS ENABLE META-LEARNING TO SOLVE CREDIT ASSIGNMENT

**Figure 1.**
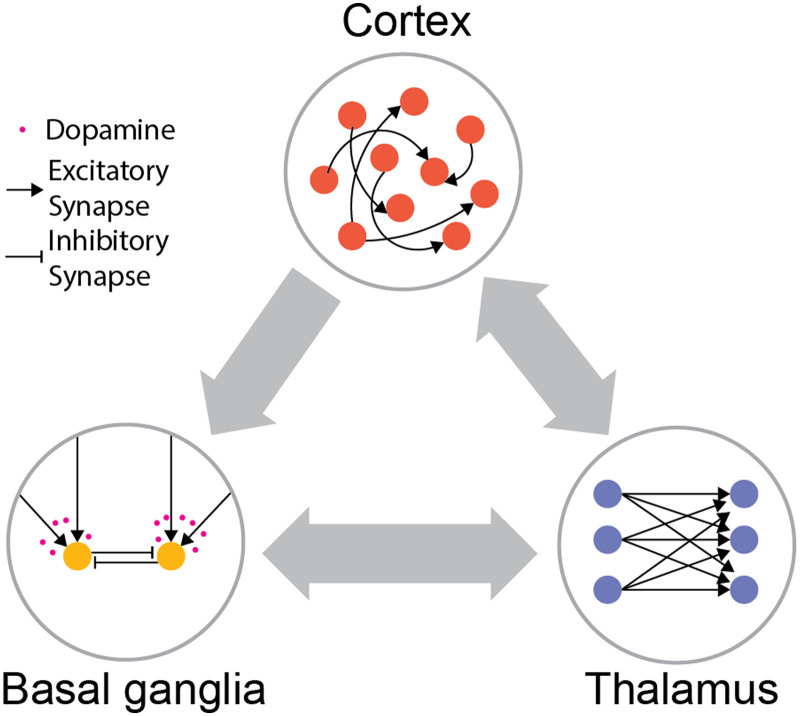
Distinct architectures of cortex, thalamus, and basal ganglia. Cortex is largely composed of excitatory neurons with extensive recurrent connectivity. Thalamus consists of mostly excitatory neurons without lateral connections. Basal ganglia consist of mostly inhibitory neurons driven by cortical and thalamic inputs, and the corticostriatal plasticity is modulated by dopamine.

To motivate the notion of thalamocortical–basal ganglia interactions being a potential solution for credit assignment, we will start with a brief introduction. The cortex, thalamus, and basal ganglia are the three major components of the mammalian forebrain—the part of the brain to which high-level cognitive capacities are attributed to (Alexander, DeLong, & Strick, 1986; Badre, Kayser, & D’Esposito, 2010; Cox & Witten, 2019; Makino, Hwang, Hedrick, & Komiyama, 2016; Miller, 2000; Miller & Cohen, 2001; Niv, 2009; Seo, Lee, & Averbeck, 2012; Wolff & Vann, 2019). Each of these components has its specialized internal architectures; the cortex is dominated by excitatory neurons with extensive lateral connectivity profiles (Fuster, 1997; Rakic, 2009; Singer, Sejnowski, & Rakic, 2019), the thalamus is grossly divided into different nuclei harboring mostly excitatory neurons devoid of lateral connections (Harris et al., 2019; Jones, 1985; Sherman & Guillery, 2005), and the basal ganglia are a series of inhibitory structures driven by excitatory inputs from the cortex and thalamus (Gerfen & Bolam, 2010; Lanciego, Luquin, & Obeso, 2012; Nambu, 2011) (Figure 1). A popular view within system neuroscience stipulates that BG and the cortex underwent different learning paradigms, where BG is involved in reinforcement learning while the cortex is involved in unsupervised learning (Doya, 1999, 2000). Specifically, the input structure of the basal ganglia known as the striatum is thought to be where reward gated plasticity takes place to implement reinforcement learning (Bamford et al., 2018; Cox & Witten, 2019; Hikosaka, Kim, Yasuda, & Yamamoto, 2014; Kornfeld et al., 2020; Niv, 2009; Perrin & Venance, 2019). One such evidence is the high temporal precision of DA activity in the striatum. To accurately attribute the action that leads to positive RPE, DA is released into the relevant corticostriatal synapses. However, DA needs to disappear quickly to prevent the next stimulus-response combination from being reinforced. In the striatum, this elimination process is carried out by dopamine active transporter (DAT) to maintain a high temporal resolution of DA activity on a timescale of around 100 ms–1 s to support reinforcement learning (Cass & Gerhardt, 1995; Ciliax et al., 1995; Garris & Wightman, 1994). In contrast, although the cortex also has dopaminergic innervation, cortical DAT expression is low and therefore DA levels may change at a timescale that is too slow to support reinforcement learning (Cass & Gerhardt, 1995; Garris & Wightman, 1994; Lapish, Kroener, Durstewitz, Lavin, & Seamans, 2007; Seamans & Robbins, 2010) but instead supports other processes related to learning (Badre et al., 2010; Miller & Cohen, 2001). In fact, ample evidence indicates that cortical structures undergo Hebbian-like long-term potentiation (LTP) and long-term depression (LTD; Cooke & Bear, 2010; Feldman, 2009; Kirkwood, Rioult, & Bear, 1996). However, despite the unsupervised nature of these processes, cortical representations are task-relevant and include appropriate sensorimotor mappings that lead to rewards (Allen et al., 2017; Donahue & Lee, 2015; Enel, Wallis, & Rich, 2020; Jacobs & Moghaddam, 2020; Petersen, 2019; Tsutsui, Hosokawa, Yamada, & Iijima, 2016). How could this arise from an unsupervised process? One possible explanation is that basal ganglia activate the appropriate cortical neurons during behaviors and the cortical network collectively consolidates high-reward sensorimotor mappings via Hebbian-like learning (Andalman & Fee, 2009; Ashby, Ennis, & Spiering, 2007; Hélie, Ell, & Ashby, 2015; Tesileanu, Olveczky, & Balasubramanian, 2017; Warren, Tumer, Charlesworth, & Brainard, 2011). Previous computational accounts of this process have emphasized a consolidation function for the cortex in this process, which naively would beg the question of why duplicate a process that seems to function well in the basal ganglia and perhaps include a lot of details of the associated experience?

**Figure 2.**
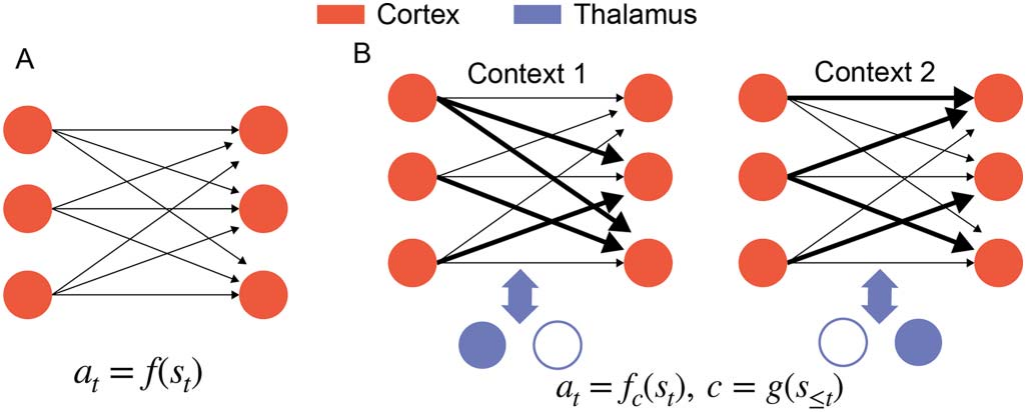
Two views of learning in the cortex. (A) One possible view is that the Hebbian cortical plasticity consolidates the sensorimotor mapping from BG to learn a stimulus-action mapping *a*_*t*_ = *f*(*s*_*t*_). (B) We propose that thalamocortical systems perform meta-learning by consolidating the teaching signals from BG to learn a context-dependent mapping *a*_*t*_ = *f*_*c*_(*s*_*t*_), where the context *c* is computed by past stimulus history and represented by different thalamic activities.

The answer to this question is the core of our proposal. We propose that the learning process is not a duplication, but instead that the reinforcement process in the basal ganglia selects thalamic control functions that subsequently activate cortical associations to allow flexible mappings across different contexts (Figure 2).

To understand this proposition, we need to take a closer look at the involvement of these distinct network elements in task learning. Learning in basal ganglia happens in corticostriatal synapses where the basic form of reinforcement learning is implemented. Specifically, the coactivation of sensory and motor cortical inputs generates eligibility traces in corticostriatal synapses that get captured by the presence or absence of DA (Fee & Goldberg, 2011; Fiete, Fee, & Seung, 2007; Kornfeld et al., 2020). This reinforcement learning algorithm is fast at acquiring simple associations but slow at generalization to other behaviors. On the other hand, the cortical plasticity operates in a much slower timescale but seems to allow flexible behaviors and fast generalization (Kim, Johnson, Cilles, & Gold, 2011; Mante, Sussillo, Shenoy, & Newsome, 2013; Miller, 2000; Miller & Cohen, 2001). How does the cortex exhibit slow synaptic plasticity and flexible behaviors at the same time? An explanatory framework is **meta-learning** (Botvinick et al., 2019; Wang et al., 2018), where the flexibility arises from network dynamics and the generalization emerges from slow synaptic plasticity across different contexts. In other words, synaptic plasticity stores a higher order association between contexts and sensorimotor associations while the network dynamics switches between different sensorimotor associations based on this higher order association. However, properly arbitrating between synaptic plasticity and network dynamics to store such higher order association is a nontrivial task (Sohn, Meirhaeghe, Rajalingham, & Jazayeri, 2021). We propose that the thalamocortical system learns these dynamics, where the thalamus provides control nodes that parametrize the cortical activity association space. Basal ganglia inputs to the thalamus learn to select between these different control nodes, directly implementing the interface between weight adjustment and dynamical controls. Our proposal rests on the following three specific points.

First, building on a line of the literature that shows diverse thalamocortical interaction in sensory, cognitive, and motor cortex, we propose that thalamic output may be described as control functions over cortical computations. These control functions can be purely in the sensory domain like attentional filtering, in the cognitive domain like manipulating working memory, or in the motor domain like preparation for movement (Bolkan et al., 2017; W. Guo, Clause, Barth-Maron, & Polley, 2017; Z. V. Guo et al., 2017; Mukherjee et al., 2020; Rikhye, Gilra, & Halassa, 2018; Saalmann & Kastner, 2015; Schmitt et al., 2017; Tanaka, 2007; Wimmer et al., 2015; Zhou, Schafer, & Desimone, 2016). These functions directly relate thalamic activity patterns to different cortical dynamical regimes and thus offer a way to establish higher order association between context and sensorimotor mapping within the thalamocortical pathways. Second, based on previous studies on direct and indirect BG pathways that influence most cortical regions (Hunnicutt et al., 2016; Jiang & Kim, 2018; Nakajima, Schmitt, & Halassa, 2019; Peters, Fabre, Steinmetz, Harris, & Carandini, 2021), we propose that BG hierarchically selects these thalamic control functions to influence activities of the cortex toward rewarding behavioral outcomes. Lastly, we propose that thalamocortical structure consolidates the selection of BG through a two-timescale Hebbian learning process to enable meta-learning. Specifically, the faster corticothalamic plasticity learns the higher order association that enables flexible contextual switching with different thalamic patterns (Marton, Seifikar, Luongo, Lee, & Sohal, 2018; Rikhye et al., 2018), while the slower cortical plasticity learns the shared representations that allow generalization to new behaviors. Below, we will go over the supporting literature that leads us to this proposal.

## MORE GENERAL ROLES OF THALAMOCORTICAL INTERACTION AND BASAL GANGLIA

Classical literature has emphasized the role of the thalamus in transmitting sensory inputs to the cortex. This is because some of the better studied thalamic pathways are those connected to sensors on one end and primary cortical areas on another (Hubel & Wiesel, 1961; Lien & Scanziani, 2018; Reinagel, Godwin, Sherman, & Koch, 1999; Sherman & Spear, 1982; Usrey, Alonso, & Reid, 2000). From that perspective, thalamic neurons being devoid of lateral connection transmit their inputs (e.g., from the retina in the case of the lateral geniculate nucleus, LGN) to the primary sensory cortex (V1 in this same example case), and the input transformation (center-surround to oriented edges) occurs within the cortex (Hoffmann, Stone, & Sherman, 1972; Hubel & Wiesel, 1962; Lien & Scanziani, 2018; Usrey et al., 2000). In many cases, these formulations of thalamic “relay” have generalized to how motor and cognitive thalamocortical interactions may be operating. However, in contrast to the classical relay view of the thalamus, more recent studies have shown diverse thalamic functions in sensory, cognitive, and motor processing (Bolkan et al., 2017; W. Guo et al., 2017; Z. V. Guo et al., 2017; Rikhye et al., 2018; Saalmann & Kastner, 2015; Schmitt et al., 2017; Tanaka, 2007; Wimmer et al., 2015; Zhou et al., 2016). For example in mice, sensory thalamocortical transmission can be adjusted based on prefrontal cortex (PFC)-dependent, top-down biasing signals transmitted through nonclassical basal ganglia pathways involving the thalamic reticular nucleus (TRN; Nakajima et al., 2019; Phillips, Kambi, & Saalmann, 2016; Wimmer et al., 2015). Interestingly, these task-relevant PFC signals themselves require long-range interactions with the associative mediodorsal (MD) thalamus to be initiated, maintained, and flexibly switched (Rikhye et al., 2018; Schmitt et al., 2017; Wimmer et al., 2015). One can also observe nontrivial control functions in the motor thalamus. Motor preparatory activities in the anterior motor cortex (ALM) show persistent activities that predicted future actions. Interestingly, the motor thalamus also shows similar preparatory activities that predict future actions and by optogenetically manipulating the motor thalamus activities, the persistent activities in ALM quickly diminished (Z. V. Guo et al., 2017). Recently, Mukherjee, Lam, Wimmer, and Halassa (2021) discovered two cell types within MD thalamus differentially modulate the cortical evidence accumulation dynamics depending on whether the evidence is conflicting or sparse to boost the signal-to-noise ratio in decision-making. Based on the above studies, we propose that the thalamus provides a set of control functions to the cortex. Specifically, cortical computations may be flexibly switched to different dynamical modes by activating a particular thalamic output that corresponds to that mode.

On the other hand, the selective role of BG in motor and cognitive control also has dominated the literature because thalamocortical–basal ganglia interaction is the most well studied in frontal systems (Cox & Witten, 2019; Makino et al., 2016; McNab & Klingberg, 2008; Monchi, Petrides, Strafella, Worsley, & Doyon, 2006; Seo et al., 2012). However, classical and contemporary studies have recognized that all cortical areas, including primary sensory areas, project to the striatum (Hunnicutt et al., 2016; Jiang & Kim, 2018; Peters et al., 2021). Similarly, the basal ganglia can project to the more sensory parts of the thalamus through lesser studied pathways to influence the sensory cortex (Hunnicutt et al., 2016; Nakajima et al., 2019; Peters et al., 2021). Specifically, a nonclassical BG pathway projects to TRN, which in turn modulates the activities of LGN to influence sensory thalamocortical transmission (Nakajima et al., 2019). On the other hand, it has also been argued that BG is involved in gating working memory (McNab & Klingberg, 2008; Voytek & Knight, 2010). This shows that BG has a much more general role than classical action and action strategy selection. Therefore, combining with our proposals on thalamic control functions, we propose that BG hierarchically selects different thalamic control functions to influence all cortical areas in different contexts through reinforcement learning.

Furthermore, there are series of the work that indicates the role of BG to guide plasticity in thalamocortical structures (Andalman & Fee, 2009; Fiete et al., 2007; Hélie et al., 2015; Mehaffey & Doupe, 2015; Tesileanu et al., 2017). In particular, there is evidence that BG is critical for the initial learning and less involved in the automatic behaviors once the behaviors are learned across different species. In zebra finches, the lesion of BG in adult zebra finch has little effect on song production, but the lesion of BG in juvenile zebra finch prevents the bird from learning the song (Fee & Goldberg, 2011; Scharff & Nottebohm, 1991; Sohrabji, Nordeen, & Nordeen, 1990). Similar patterns can be observed in people with Parkinson’s disease. Parkinson’s patients who have a reduction of DA and striatal defects have troubles in solving procedural learning tasks but can produce automatic behaviors normally (Asmus, Huber, Gasser, & Schöls, 2008; Soliveri, Brown, Jahanshahi, Caraceni, & Marsden, 1997; Thomas-Ollivier et al., 1999). This behavioral evidence suggests that thalamocortical structures consolidate the learning from BG as the behaviors become more automatic. Furthermore, on the synaptic level, a songbird learning circuit also demonstrates this cortical consolidation motif (Mehaffey & Doupe, 2015; Tesileanu et al., 2017). In a zebra finch, the premotor nucleus HVC (a proper name) projects to the motor nucleus robust nucleus of the arcopallium (RA) to produce the song. On the other hand, RA also receives BG nucleus Area X mediated inputs from the lateral nucleus of the medial nidopallium (LMAN). The latter pathway is believed to be a locus of reinforcement learning in the songbird circuit. By burst stimulating both input pathways in different time lags, one can discover that HVC-RA and LMAN-RA underwent opposite plasticity (Mehaffey & Doupe, 2015). This suggests that the learning is gradually transferred from LMAN-RA to HVC-RA pathway (Fee & Goldberg, 2011; Mehaffey & Doupe, 2015; Tesileanu et al., 2017). This indicates a general role of BG as the trainer for cortical plasticity.

## THE THALAMOCORTICAL STRUCTURE CONSOLIDATES THE BG SELECTIONS ON THALAMIC CONTROL FUNCTIONS IN DIFFERENT TIMESCALES TO ENABLE META-LEARNING

In this section, in addition to BG’s role as the trainer for cortical plasticity, we further propose that BG is the trainer in two different timescales for thalamocortical structures to enable meta-learning. The faster timescale trainer trains the corticothalamic connections to select the appropriate thalamic control functions in different contexts, while the slower timescale trainer trains the cortical connections to form a task-relevant and generalizable representation.

**Figure 3.**
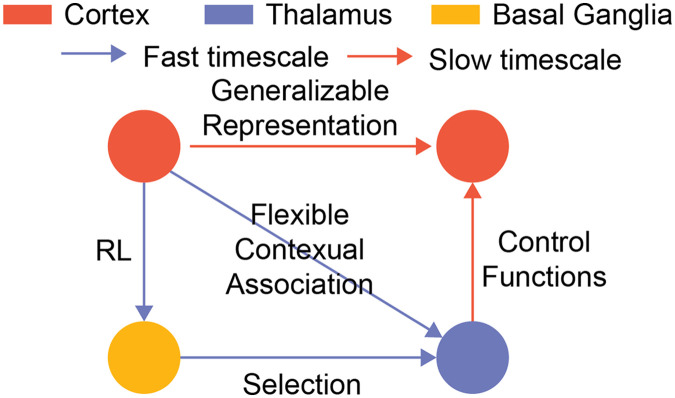
Two-timescale learning in thalamocortical structures. We propose that one can learn the thalamocortical structure to enable meta-learning by applying the general network motif in two different timescales. First, one can learn the corticothalamic connections by applying the motif on the blue loop with a faster timescale. This allows the network to consolidate flexible switching behaviors. Second, one can learn the cortical connections by applying the motif on the orange loop in a slower timescale. This allows cortical neurons to develop a task-relevant shared representation that can generalize across contexts.

From the songbird example, we see how thalamocortical structures can consolidate simple associations learned through the basal ganglia. To enable meta-learning, we propose that this general network consolidation motif operates over two different timescales within thalamocortical–basal ganglia interactions (Figure 3). First, combining the idea of thalamic outputs as control functions over cortical network activity patterns and the basal ganglia selecting such functions, we frame learning in basal ganglia as a process that connects contextual associations (higher order) with the appropriate dynamical control that maximizes reward at the sensorimotor level (lower order). Under this framing, corticothalamic plasticity consolidates the higher order association within a fast timescale. This allows flexible switching between different thalamic control functions in different contexts. On the other hand, the cortical plasticity consolidates the sensorimotor association over a slow timescale to allow shared representation that can generalize across different contexts. As the thalamocortical structures learn the higher order association, the behaviors become less BG-dependent and the network is able to switch between different thalamic control functions to induce different sensorimotor mappings in different contexts. By having two learning timescales, animals can conceivably both adapt quickly in changing environments with fast learning of corticothalamic connections and maintain the important information across the environment in the cortical connections. One should note that this separation of timescales is independent from different timescales across cortex (Gao, van den Brink, Pfeffer, & Voytek, 2020; J. D. Murray et al., 2014). While different timescales across cortex allows animals to process information differentially, the separation of corticothalmic and cortical plasticity allows the thalamocortical system to learn the higher contextual association to modulate cortical dynamics flexibly.

Some anatomical observations support this idea. The thalamostriatal neurons have a more modulatory role to the cortical dynamics in a diffusive projection, while thalamocortical neurons have a more driver role to the cortical dynamic in a topographically restricted dense projection (Sherman & Guillery, 2005). This indicates that thalamostriatal neurons might serve as the role of control functions in the faster consolidation loop with the feedback to striatum to conduct credit assignment. On the other hand, thalamocortical neurons might be more involved in the slower consolidation loop with the feedback to striatum coming from the cortex to train the common cortical representation across contexts.

In summary, this two-timescale network consolidation scheme provides a general way for BG to guide plasticity in the thalamocortical architecture to enable meta-learning and thus solves structural credit assignment as a special case. Along these lines, experimental evidence supports the notion that when faced with multisensory inputs, the BG can selectively disinhibit a modality-specific subnetwork of the thalamic reticular nucleus (TRN) to filter out the sensory inputs that are not relevant to the behavior outcomes and thus solve the structural credit assignment problem.

In the discussion above, we discuss our proposal under a general formulation of thalamic control functions. In the next section, we will specify other thalamic control functions suggested by recent studies and observe how they can solve continual learning under this framework as well.

## THE THALAMUS SELECTIVELY AMPLIFIES FUNCTIONAL CORTICAL CONNECTIVITY AS A SOLUTION TO CONTINUAL LEARNING AND CATASTROPHIC FORGETTING

One of the pitfalls of the artificial neural network is catastrophic forgetting. If one trains an artificial neural network on a sequence of tasks, the performance on the older task will quickly deteriorate as the network learns the new task (French, 1999; Kemker et al., 2018; Kumaran et al., 2016; McCloskey & Cohen, 1989; Parisi et al., 2019). On the other hand, the brain can achieve *continual learning*, the ability to learn different tasks in different contexts without catastrophic forgetting and even generalize the performance to novel context (Lewkowicz, 2014; M. M. Murray et al., 2016; Power & Schlaggar, 2017; Zenke, Gerstner, & Ganguli, 2017). There are three main approaches in machine learning to deal with catastrophic forgetting. First, one can use the regularization method to mostly update the weights that are less important to the prior tasks (Fernando et al., 2017; Jung, Ju, Jung, & Kim, 2018; Kirkpatrick et al., 2017; Li & Hoiem, 2018; Maltoni & Lomonaco, 2019; Zenke, Poole, & Ganguli, 2017). This idea is inspired by experimental and theoretical studies on how synaptic information is selectively protected in the brain (Benna & Fusi, 2016; Cichon & Gan, 2015; Fusi, Drew, & Abbott, 2005; Hayashi-Takagi et al., 2015; Yang, Pan, & Gan, 2009). However, it is unclear how to biologically compute the importance of each synapse to prior tasks nor how to do global regularization locally. Second, one can also use a dynamic architecture in which the network expands the architecture by allocating a subnetwork to train with the new information while preserving old information (Cortes, Gonzalvo, Kuznetsov, Mohri, & Yang, 2017; Draelos et al., 2017; Rusu et al., 2016; Xiao, Zhang, Yang, Peng, & Zhang, 2014). However, this type of method is not scalable since the number of neurons needs to scale linearly with the number of tasks. Lastly, one can use a memory buffer to replay past tasks to avoid catastrophic forgetting by interleaving the experience of the past tasks with the experience of the present task (Kemker & Kanan, 2018; Kumaran et al., 2016; McClelland et al., 1995; Shin, Lee, Kim, & Kim, 2017). However, this type of method cannot be the sole solution, as the memory buffer needs to scale linearly with the number of tasks and potentially the number of trials.

**Figure 4.**
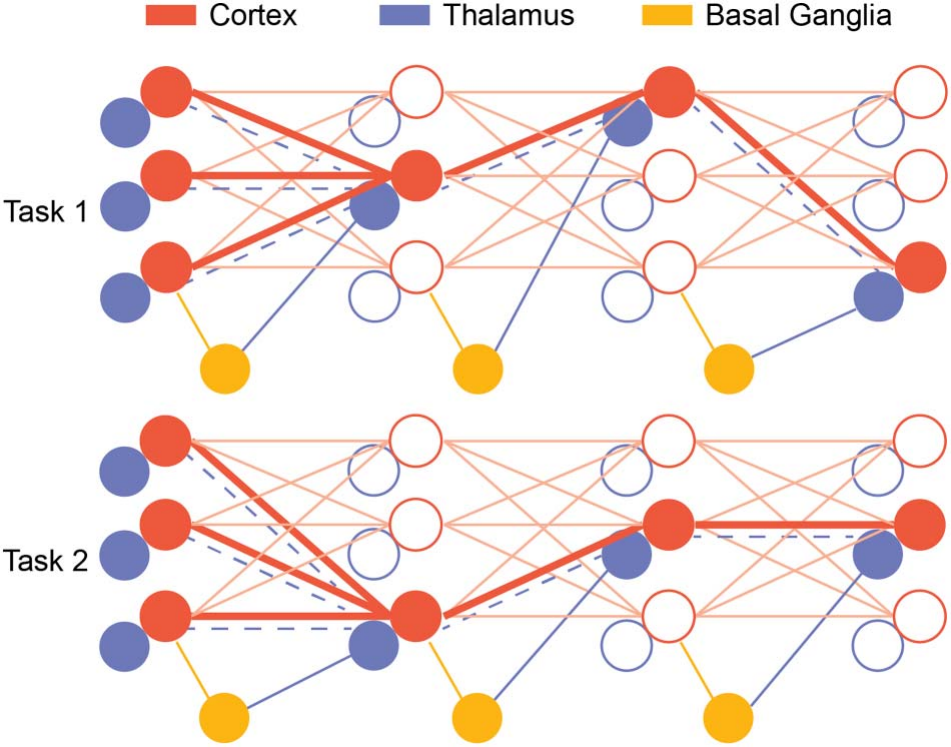
A thalamocortical architecture with interaction with BG for continual learning. During task execution, BG selects thalamic neurons that amplify the relevant cortical subnetwork. This protects other parts of the network that are important for another context from being overwritten. When the other task comes, BG selects other thalamic neurons and since the synapses are protected from the last task, animals can freely switch from different tasks without forgetting the previous tasks. Furthermore, as the corticothalamic synapses learn how to select the right thalamic neurons in a different context (blue dashed line), task execution can become less BG dependent.

We propose that the thalamus provides another way to solve continual learning and catastrophic forgetting via selectively amplifying parts of the cortical connections in different contexts (Figure 4). Specifically, we propose that a population of thalamic neurons topographically amplify the connectivity of cortical subnetworks as their control functions. During a behavioral task, BG selects subsets of the thalamus that selectively amplify the connectivity of cortical subnetworks. Because of the reinforcement learning in BG, the subnetwork that is the most relevant to the current task will be more preferentially activated and updated. By selecting only the relevant subnetwork to activate in one context, the thalamus protects other subnetworks that can have useful information in another context from being overwritten. The corticothalamic structures can then consolidate these BG-guided flexible switching behaviors via our proposed network motif, and the switching becomes less BG-dependent. Furthermore, our proposed solution has implications on generalization as well. Different tasks can have principles in common that can be transferred. For example, although the rules of chess and Go are very different, players in both games all need to predict what the other players are going to do and counterattack based on the prediction. Since BG selects the subnetwork at each hierarchy that is most relevant to the current tasks, in addition to selecting different subnetworks to prevent catastrophic forgetting, BG can also select subnetworks that are beneficial to both tasks as well to achieve generalization. Therefore, the cortex can develop a modular hierarchical representation of the world that can be easily generalized.

The idea of protecting relevant information from the past tasks to be overwritten has been applied before computationally and has decent success in combating catastrophic forgetting in deep learning (Kirkpatrick et al., 2017). Experimentally, we also have found that thalamic neurons selectively amplify the cortical connectivity to solve the continual learning problem. In a task where the mice need to switch between different sets of task cues that guided the attention to the visual or auditory target, the performance of the mice does not deteriorate much after switching to the original context, which is an indication of continual learning (Rikhye et al., 2018). Through electrophysiological recording of PFC and mediodorsal thalamic nucleus (MD) neurons, we discovered that PFC neurons preferentially code for the rule of the attention, while MD neurons preferentially code for the contexts of different sets of the cues. Thalamic neurons that encode the task-relevant context translate this neural representation into the amplification of cortical activity patterns associated with that context (despite the fact that cortical neurons themselves only encode the context implicitly). These experimental observations are consistent with our proposed solution: By incorporating the thalamic population that can selectively amplify connectivity of cortical subnetworks, the thalamus and its interaction with cortex and BG solve the continual learning problem and prevent catastrophic forgetting.

## CONCLUSION

In summary, in contrast to the traditional relay view of the thalamus, we propose that thalamocortical interaction is the locus of meta-learning where the thalamus provides cortical control functions, such as sensory filtering, working memory gating, or motor preparation, that parametrize the cortical activity association space. Furthermore, we propose a two-timescale learning consolidation framework in which BG hierarchically selects these thalamic control functions to enable meta-learning, solving the credit assignment problem. The faster plasticity learns contextual associations to enable rapid behavioral flexibility, while the slower plasticity establishes cortical representation that generalizes. By considering the recent observation of the thalamus selectively amplifying functional cortical connectivity, the thalamocortical–basal ganglia network is able to flexibly learn context-dependent associations without catastrophic forgetting while generalizing to the new contexts. This modular account of the thalamocortical interaction may seem to be in contrast with the recent proposed dynamical perspectives (Barack & Krakauer, 2021) on thalamocortical interaction in which the thalamus shapes and constrains the cortical attractor landscapes (Shine, 2021). We would like to argue that both the modular and the dynamical perspectives are compatible with our proposal. The crux of the perspectives is that the thalamus provides control functions that parametrize cortical dynamics, and these control functions can be of modular nature or of dynamical nature depending on their specific input-output connectivity. Flexible behaviors can be induced by selecting either the control functions that amplify the appropriate cortical subnetworks or those that adjust the cortical dynamics to the appropriate regimes.

## AUTHOR CONTRIBUTIONS

Mien Wang: Conceptualization; Investigation; Methodology; Writing – original draft; Writing – review & editing. Michael M. Halassa: Conceptualization; Funding acquisition; Methodology; Supervision; Writing – review & editing.

## FUNDING INFORMATION

Michael M. Halassa, National Institute of Mental Health (https://dx.doi.org/10.13039/100000025), Award ID: 5R01MH120118-02.

## TECHNICAL TERMS

Reward prediction error: A quantity represented by the difference between the expected reward and actual reward.
Credit assignment: A computational problem to determine which stimulus, action, internal states, and context lead to outcome.
Continual learning: A computational problem to learn tasks sequentially to both learn new tasks faster and not forget old tasks.
Backpropagation: An algorithm to compute the error gradient of an artificial neural network through chain rules.
Catastrophic forgetting: A phenomenon in which the network forgets about the previous tasks upon learning new tasks.
Meta-learning: A learning paradigm in which a network learns how to learn more efficiently.
